# Separation of highly hydrophilic nicotinamide metabolites using a COSMOSIL PBr column

**DOI:** 10.1016/j.mex.2023.102061

**Published:** 2023-02-08

**Authors:** Makoto Ozaki, Motoshi Shimotsuma, Tsunehisa Hirose

**Affiliations:** Nacalai Tesque, Inc., Ishibashi Kaide-cho, Muko-shi, Kyoto 617-0004, Japan

**Keywords:** Nicotinamide mononucleotide (NMN), Reversed phase chromatography, HPLC, LC-MS, and LC-MS/MS analysis, Separation of nicotinamide metabolites in foods

## Abstract

Highly hydrophilic compounds such as nicotinamide metabolites are very difficult to separate via high-performance liquid chromatography (HPLC) using octadecyl (C_18_) columns. In general, for the separation of hydrophilic compounds, hydrophilic interaction liquid chromatography (HILIC) columns are used instead of reversed phase chromatography using C_18_ columns. However, HILIC columns generally obey complex separation mechanisms because ionic interactions are involved in the retention process, which hinders the optimization of the separation conditions. Additionally, the resulting peak shapes are disturbed when large amounts of aqueous samples are injected. This study demonstrates that COSMOSIL PBr columns, in which both hydrophobic and dispersive interactions occur, show high retention for various hydrophilic compounds under similar separation conditions as those used with C_18_ columns. Specifically, using a COSMOSIL PBr column, 11 nicotinamide metabolites could be separated under simpler conditions than those used previously with C_18_ columns, affording better peak shape for each compound. The applicability of the method was evaluated using a tomato sample, from which the nicotinamide metabolites were successfully separated. The results show that the COSMOSIL PBr column is a good alternative to the C_18_ column for a good separation of all the peaks, including impurity peaks.

## Graphical abstract

**Figure:**
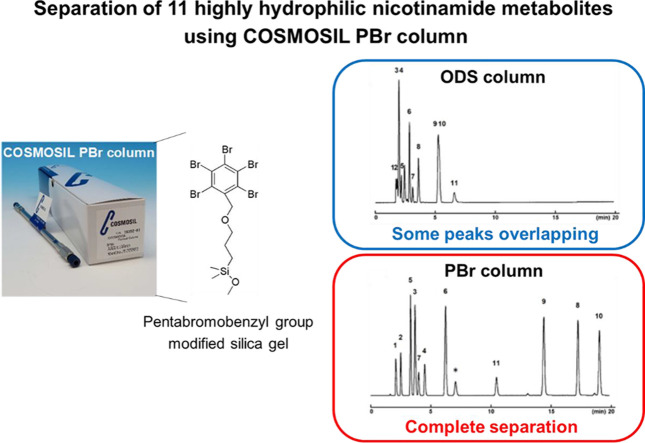

Specifications table

**Table:** 

Subject Area	Biochemistry, Genetics and Molecular Biology

More specific subject area:	Metabolites analysis
Name of your method:	Separation of nicotinamide metabolites in foods
Protocol name:	Separation of highly hydrophilic nicotinamide metabolites under simple separation condition using a COSMOSIL PBr column
Reference of original method:	M. Ozaki, M. Shimotsuma, T. Hirose, Separation of nicotinamide metabolites using a PBr column packed with pentabromobenzyl group modified silica gel, Anal. Biochem. 655 (2022) 114837.
Reagents/tools:	Nicotinic acid mononucleotide (NAMN) Nicotinamide mononucleotide (NMN) Adenosine triphosphate (ATP) Inosinic acid (IMP) Nicotinamide riboside (NR) Cytidine Nicotinic acid (NA) Nicotinamide adenine dinucleotide in oxidized form (NAD^+^) Inosine Nicotinamide adenine dinucleotide in reduced form (NADH) Nicotinamide (NAM)
	Methanol Ammonium formate Chloroform COSMOSIL 3PBr (3.0 mm I.D. × 150 mm, particle size; 3 µm, Nacalai Tesque, Kyoto, Japan) SHIMADZU HPLC system equipped with an LC-30 AD intelligent pump (Shimadzu, Kyoto, Japan) LTQ XL mass spectrometer (Thermo Scientific, Massachusetts, U.S.A.)
Experimental design:	We developed a method for the separation of highly hydrophilic nicotinamide metabolites under simple conditions using a COSMOSIL PBr column. COSMOSIL PBr columns are used in reversed phase mode like typical octadecyl (C_18_) columns, but they can separate various hydrophilic compounds that are difficult to separate with C_18_ columns. Our separation method using a COSMOSIL PBr column allows performing a liquid chromatography–mass spectrometry (LC–MS) analysis because ammonium formate is used as the mobile phase. Moreover, this method outperforms other previous studies in terms of the peak shape.
Trial registration:	N/A
Ethics:	N/A
Value of the Protocol:	• Our proposed method requires little to no optimization time. • The method allows the baseline separation of 11 highly hydrophilic nicotinamide metabolites that are difficult to separate on a C_18_ column under simple conditions. • Good peak shapes are obtained for nicotinamide metabolites.

## Description of protocol

**Fig. 1:**
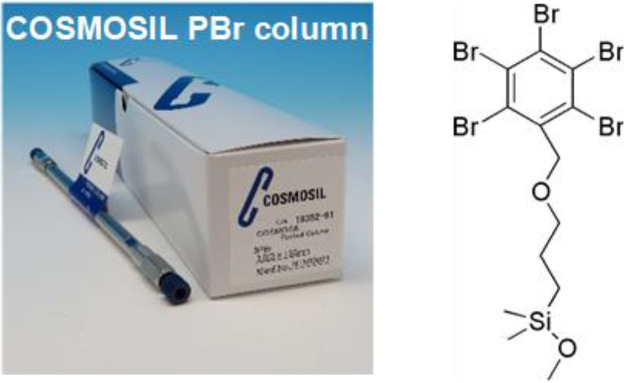
Structure of the stationary phase of COSMOSIL PBr column.

Nicotinamide metabolites such as NAD^+^ and NMN are involved in a variety of biological processes, such as the prevention of aging and cancer. Therefore, the development of methods based on HPLC for the separation and identification of nicotinamide metabolites is important. However, nicotinamide metabolites are highly hydrophilic and poorly retained on C_18_ columns. In contrast, COSMOSIL PBr columns packed with a stationary phase similar to 3-(pentabromobenzyloxy)propyl (Fig. 1) provide high retention for various hydrophilic compounds because of the action of hydrophobic interactions and dispersion forces. Therefore, 11 nicotinamide metabolites were separated under simple conditions using a COSMOSIL PBr column. As a proof-of-concept, nicotinamide metabolites were separated and identified from a tomato sample.

## Methods details

### Chemicals

Nicotinic acid mononucleotide (NAMN; Cat. No. 32883) was purchased from Funakoshi Co., Ltd. (Tokyo, Japan). NMN (Cat. No. N1123) and cytidine (Cat. No. C0522) were purchased from Tokyo Chemical Industries (Tokyo, Japan). Adenosine triphosphate (ATP; Cat. No. 01072–11), nicotinic acid (NA; Cat. No. 24326–52), nicotinamide (NAM; Cat. No. 24317–72), inosinic acid (IMP; Cat. No. 06400–51), inosine (Cat. No. 07139–42), nicotinamide adenine dinucleotide in oxidized form (NAD^+^; Cat. No. 24338–86), nicotinamide adenine dinucleotide in reduced form (NADH; Cat. No. 24335–61), methanol (HPLC grade, Cat. No. 21929–23), chloroform (HPLC grade, Cat. No. 08426–13), and ammonium formate (guaranteed reagent, Cat. No. 02509–55) were obtained from Nacalai Tesque Inc. (Kyoto, Japan). Nicotinamide riboside chloride (NR; Cat. No. A220128) was purchased from Cosmo Bio Co., Ltd. (Tokyo, Japan).

### Sample preparation (liquid–liquid extraction of nicotinamide metabolites in tomato)

A tomato sample (1 g) was suspended in 2 mL of chloroform/methanol (1/2, vol./vol.) and 400 µL of ultrapure water. After centrifugation at 9100 rpm for 10 min (room temperature), 2 mL of the supernatant was transferred into an eggplant flask and dried by evaporation. Subsequently, the residue was dissolved with 200 µL of ultrapure water.

### Preparation of the mobile phase

A 20 mM ammonium formate solution (pH 6.4) was prepared by dissolving 1.26 g of ammonium formate powder (M.W. 63.06 g/mol) in ultrapure water and filling up to 1 L (no need to adjust pH). The solution was suction-filtered using MILLICUP-HV filter units (pore size; 0.45 µm, Cat. No. SJHVM4710, Merck, Darmstadt, Germany).

Solvent A (500 mL) was prepared by mixing 475 mL of the 20 mM ammonium formate solution (pH 6.4) with 25 mL of methanol.

Solvent B (500 mL) was prepared by mixing 400 mL of the 20 mM ammonium formate solution (pH 6.4) with 100 mL of methanol.

### Preparation for HPLC analysis

After starting up the SHIMADZU HPLC system equipped with an LC-30 AD intelligent pump and an SPD-20AD detector (Shimadzu, Kyoto, Japan), the feed line was changed from the wash solution (ultrapure water/methanol, 1/1, vol./vol.) to solvent A and B. To prevent the wash solution and the mobile phase from mixing, solution was removed from the feed line, and a small amount of air was introduced into the feed line before putting the feed line into the mobile phase. After purging, the COSMOSIL 3PBr column (3.0 mm I.D. × 150 mm, particle size; 3 µm, Cat. No. 19352–91) was attached and equilibrated with 100% solvent A at a temperature of 40 °C and a flow rate of 0.4 mL/min for 10–15 min. A blank analysis should be performed before analyzing the sample. Each standard sample (except NAD^+^) was dissolved in ultrapure water to 5 mM (NAD^+^ was dissolved in 1 M NaOH to 5 mM). The peak shape and retention time of each standard sample should be confirmed by HPLC analysis before analyzing nicotinamide metabolites in foods.

### Preparation for LC–MS analysis

An LTQ XL mass spectrometer (Thermo Scientific, Waltham, MA, USA) was used. The sheath gas nitrogen flow rate was set at 15 (arb), the aux gas flow rate was set at 5 (arb), and the block heater in the LC–MS equipment was heated to 400 °C. A mass range of *m/z* 50–1000 was covered with a scan time of 1.0 s, and data were collected in positive ion mode using a detector voltage of 1.5 kV. Nicotinamide metabolites were detected at *m/z* 336.5 (NAMN, M.W. 335.2), *m/z* 335.5 (NMN, M.W. 334.2), *m/z* 525.5 (ATP, M.W. 507.5, the *m/z* value corresponds to the ammonium ion adduct), *m/z* 124.5 (NA, M.W. 123.1), *m/z* 349.5 (IMP, M.W. 348.2), *m/z* 256.5 (NR, M.W. 255.5), *m/z* 244.5 (cytidine, M.W. 243.2), *m/z* 123.5 (NAM, M.W. 122.1)*, m/z* 269.5 (inosine, M.W. 268.2)*, m/z* 664.5 (NAD^+^, M.W. 663.4), and *m/z* 665.5 (NADH, M.W. 664.4)*.*

### HPLC and LC–MS analysis conditions

The gradient condition for the separation of nicotinamide metabolites was as follows:

Solvent B concentration (B conc.);
0% (at 0 min)
0% (at 5 min)
90% or 100% (at 20 min)
0% (at 20.1 min)
0% (at 30 min)

The analytical run proceeded in the 0–20 min time interval, and a reequilibration process was performed in the 20.1–30 min time interval.

## Representative results

**Fig. 2:**
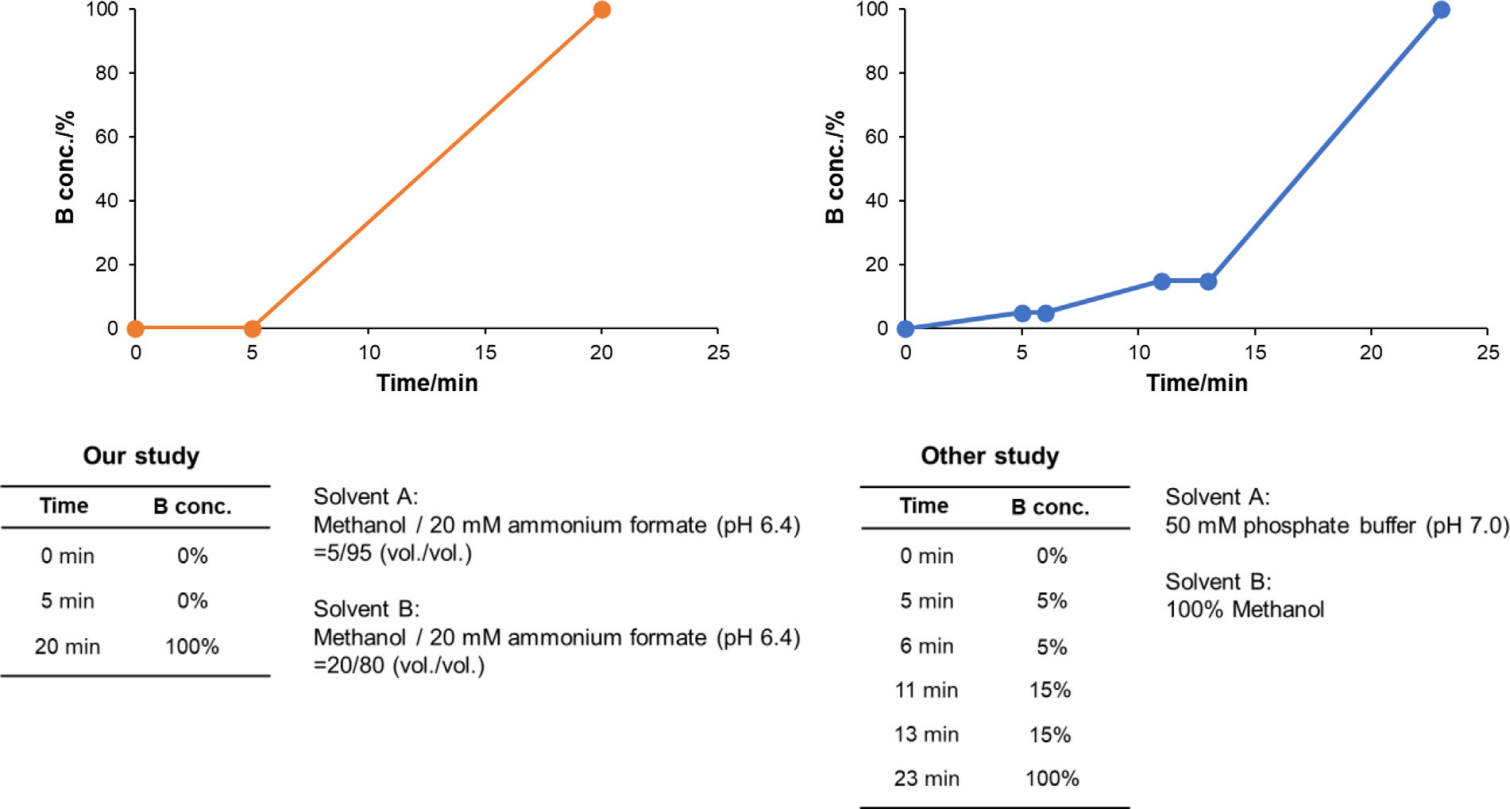
Gradient curves obtained in our study using a COSMOSIL 3PBr column (3.0 mm I.D. × 150 mm, particle size; 3 µm, Nacalai Tesque) and in a previous study using a SUPELCOSIL™ LC18-T column (4.6 mm I.D. × 150 mm, particle size; 3 µm, Sigma-Aldrich).

**Fig. 3:**
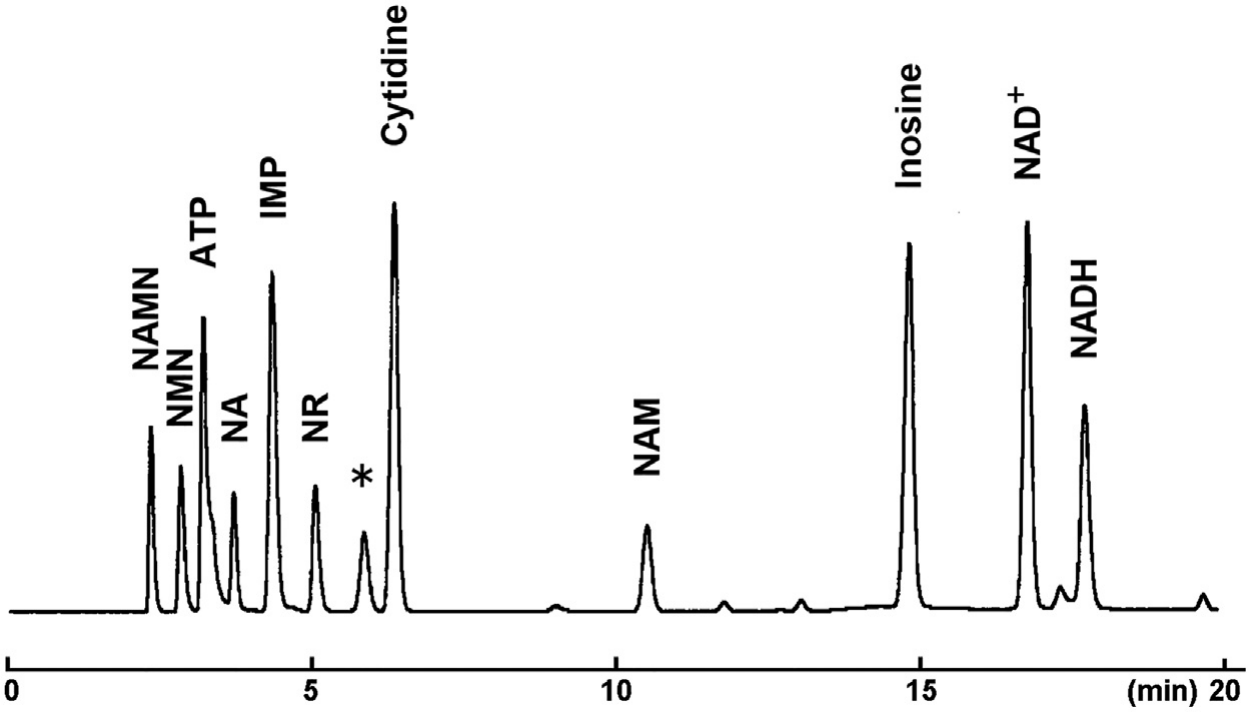
HPLC chromatogram of 11 nicotinamide metabolites (sample concentration; 250 µM, injection volume; 1.0 µL) obtained using a COSMOSIL 3PBr column (3.0 mm I.D. × 150 mm, particle size; 3 µm) with a flow rate of 0.4 mL/min at 40 ºC. UV detection was performed at 260 nm. * Peak of impurities derived from NAD^+^.

In previous studies, nicotinamide metabolites were separated under complex gradient conditions using C_18_ columns [1], [2]. However, the effects of equipment, length of piping, and mixer capacity of the complicated condition affect the reproducibility of the results (Fig. 2). In contrast, in the present study, 11 nicotinamide metabolites could be baseline-separated under simple gradient conditions using a COSMOSIL 3PBr column (Figs. 2 and 3) [3]. Additionally, the peak shape of each compound was better than in previous studies [1], [2].

**Fig. 4:**
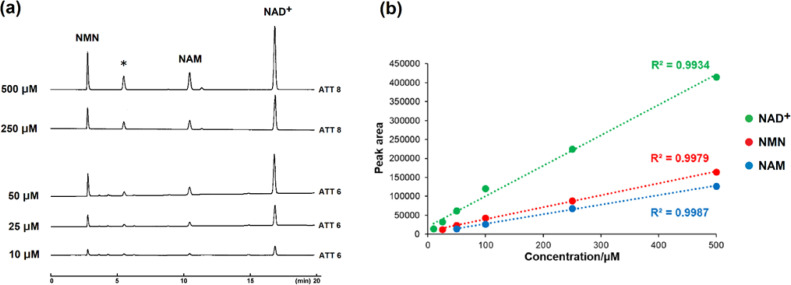
(a) HPLC traces of various concentrations of nicotinamide mononucleotide (NMN), nicotinamide (NAM), and nicotinamide adenine dinucleotide (NAD^+^) separated using a COSMOSIL 3PBr column (3.0 mm I.D. × 150 mm, particle size; 3 µm) with a flow rate of 0.4 mL/min at 40 ºC. UV detection was performed at 260 nm. * Peak of impurities derived from NAD^+^. (b) Calibration curves of NMN, NAM, and NAD^+^.

The peak shape of obtained by HPLC analysis may be disturbed depending on the type and concentration of the target substance. Therefore, the effect of a gradual decrease in the concentration on the peak shapes of NMN, NAM, and NAD^+^, which are important compounds in the salvage pathway, was investigated. No significant change in the peak shape was observed in the HPLC traces of NMN, NAM, and NAD^+^ when the concentration was decreased from 500 to 10 µM (Fig. 4a). Additionally, linear calibration curves that correlate the HPLC peak area with the concentration were constructed for NMN, NAM, and NAD^+^ (Fig. 4b).

**Fig. 5:**
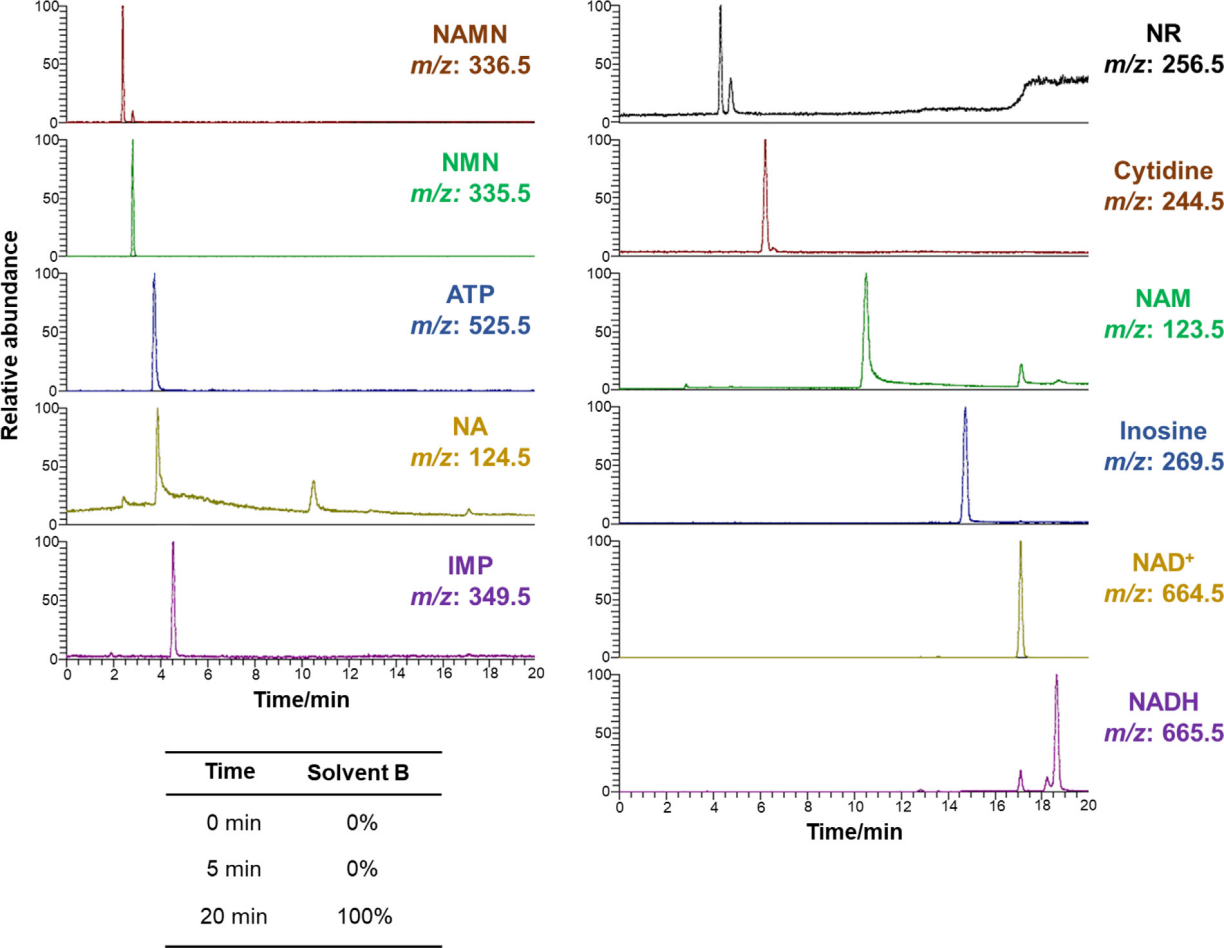
LC–MS chromatograms of 11 nicotinamide metabolites separated using a COSMOSIL 3PBr column (3.0 mm I.D. × 150 mm, particle size; 3 µm) with a flow rate of 0.4 mL/min at 40 ºC.

LC–MS measurements were performed under the same separation conditions using the COSMOSIL PBr column to separate and identify 11 nicotinamide metabolites (Fig. 5). The LC–MS chromatograms showed that the peaks derived from NAMN (M.W. 335.5) and NMN (M.W. 334.5) were separated, and good peak shapes were obtained for nicotinamide metabolites.

**Fig. 6:**
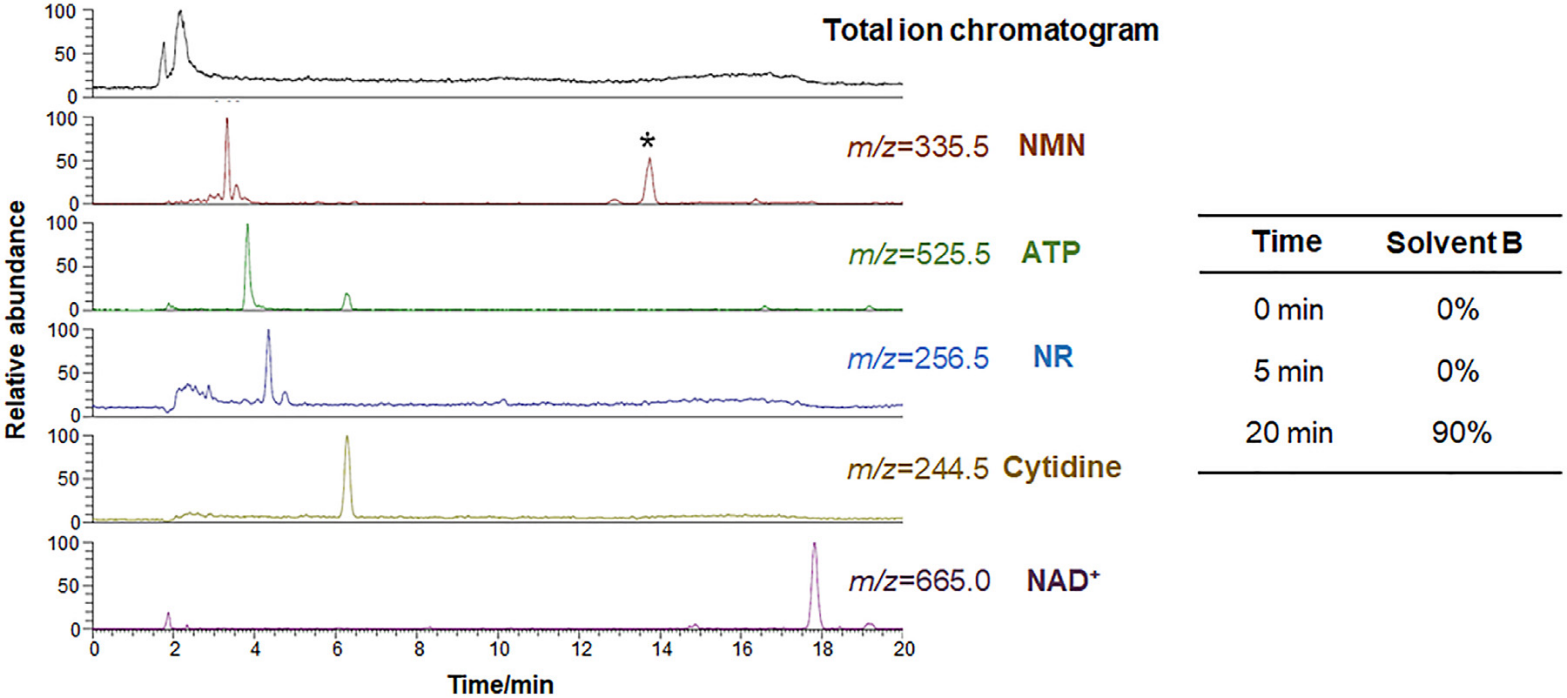
LC–MS chromatograms of nicotinamide metabolites in tomato separated using a COSMOSIL 3PBr (3.0 mm I.D. × 150 mm, particle size; 3 µm) with a flow rate of 0.4 mL/min at 40 ºC. * Other compounds than NMN.

Finally, the method was applied to the analysis of nicotinamide metabolites in tomato, which is rich in NMN [4]. After crushing the tomatoes to obtain a homogeneous sample, the sample was subjected to liquid–liquid extraction to remove proteins and lipids before the LC–MS measurements. The obtained LC–MS chromatograms showed peaks derived from NMN, ATP, NR, cytidine, and NAD^+^ (Fig. 6). Additionally, the total ion chromatogram showed that the peak of NMN was successfully separated from peaks of impurities around t_0_ (Fig. 6).

## Conclusion

We have developed a method using a COSMOSIL PBr column that enables the separation and identification of 11 nicotinamide metabolites that are difficult to separate on C_18_ columns. For all compounds, the peak shapes were better than those obtained in previous studies. Furthermore, the COSMOSIL PBr column allowed separating and identifying nicotinamide metabolites in tomatoes containing impurities via LC–MS measurements. We believe that this method can be extended to the analysis of other food samples such as avocado and broccoli and can contribute to the development of antiaging and anticancer strategies.

## CRediT authorship contribution statement

**Makoto Ozaki:** Conceptualization, Investigation, Methodology, Formal analysis, Validation, Writing – original draft, Writing – review & editing. **Motoshi Shimotsuma:** Funding acquisition, Project administration, Writing – review & editing. **Tsunehisa Hirose:** Conceptualization, Funding acquisition, Project administration, Validation, Writing – review & editing.

## Declaration of Competing Interest

The authors declare that they have no known competing financial interests or personal relationships that could have appeared to influence the work reported in this paper.

## Data Availability

Data will be made available on request.

## References

[1] Yoshio J., Imai S. (2013). Accurate measurement of nicotinamide adenine dinucleotide (NAD^+^) with high-performance liquid chromatography. Methods Mol. Biol..

[2] Trammel S., Brenner C. (2013). Targeted, LCMS-based metabolomics for quantitative measurement of NAD^+^ metabolites. Comput. Struct. Biotechnol. J..

[3] Ozaki M., Shimotsuma M., Hirose T. (2022). Separation of nicotinamide metabolites using a PBr column packed with pentabromobenzyl group modified silica gel. Anal. Biochem..

[4] Mills K.F., Yoshida S., Stein L.R., Grozio A., Kubota S., Sasaki Y., Redpath P., Migaud M.E., Apte R.S., Uchida K., Yoshino J., Imai S. (2016). Long-term administration of nicotinamide mononucleotide mitigates age-associated physiological decline in mice. Cell Metab..

